# Complex case of acute mesenteric ischemia in a patient with atrial fibrillation: A radiologic -surgical collaboration for timely intervention

**DOI:** 10.6026/9732063002001922

**Published:** 2024-12-31

**Authors:** Pritika Gnanasekaran, Navaneeth Ranjith, Devta Viswam, Kaushik Rajavel, Pavithra Rekha Ravikumar

**Affiliations:** 1 Department of Medicine, Global Medical Centre, Salem, Tamil Nadu, India; 2 Department of Urology, Caritas Hospital, Kottayam, Kerala, India; 3 Department of Radiology, Caritas Hospital, Kottayam, Kerala, India; 4 Department of Orthopaedics, Madras Medical College, Chennai, Tamil Nadu, India; 5 Department of Obstetrics & Gynaecology, Madras Medical College, Chennai, Tamil Nadu, India

**Keywords:** Acute mesenteric ischemia, atrial fibrillation, CT angiography, embolic events, surgical intervention, radiology

## Abstract

Acute mesenteric ischemia (AMI), often caused by embolic events in patients with atrial fibrillation (AF), can lead to bowel necrosis without timely intervention. This study evaluated the role of CT angiography (CTA) in diagnosing AMI and its impact on surgical outcomes through radiologic-surgical collaboration in 100 AF patients. CTA demonstrated a high diagnostic accuracy, correctly identifying AMI in 92% of cases (p < 0.001) and facilitated early surgical intervention in 75% of patients, achieving an 80% success rate. Delayed interventions were associated with increased morbidity and mortality (p = 0.003). These findings highlight the effectiveness of CTA in diagnosing AMI and underscore the importance of timely radiologic-surgical collaboration for optimal patient outcomes.

## Background:

Acute mesenteric ischemia is one of the increasingly important surgical emergencies that is associated with sudden and clinically significant diminution or total cessation of blood supply to the intestines, leading to bowel ischemia. The condition is seriously threatening at the sites involved for necrosis [1]. The usual causes of AMI include arterial embolisms as well as formation of thrombosis and non-occlusive mesenteric ischemia that lead to this deadly situation. AF is one of the frequent cardiac arrhythmias that put the patient significantly at risk for the danger of embolic events due to the predisposition of the patient to form thrombi in the heart, which may travel down to the mesenteric arteries [2]. Generalized symptoms attributed to AMI acute mesenteric ischemia, such as severe pain in the abdomen, continuous nausea and vomiting spasms, will always feature in the past with significant diagnostic delays thus compounding severe challenges on the outcome of such patients [3]. It has traditionally been diagnosed based on the clinical suspicion and procedures of exploratory laparotomy, but this has many shortcomings in that most the results are false negatives or delayed detection of the problem especially at the preliminary stages of ischemia when prompt intervention is important. The last few years, however and CTA, an acronym for CT angiography, has remained the gold standard in diagnosing AMI because it does a very efficient job of offering perfectly accurate visualization of mesenteric vessels that can be helpful in identifying arterial occlusions that might be causing the ischemic condition [4]. A specific place in discussions about complex management of acute myocardial infarction in patients with atrial fibrillation is a special role of CTA in the early detection and cooperation partnership between radiologists and surgeons for a timely performance of intervention [5]. Therefore, it is of interest to the clinical cohort of patients with AF complicated by AMI to be implanted in the strategy formulating that aims for improvements of the diagnostic accuracy and survival in the patient [6].

## Methodology:

This is a retrospective study between January 2022 and December 2023 involving a total of 100 patients with acute mesenteric ischemia secondary to atrial fibrillation. The sensitivity of the ability for CTA to diagnose AMI was calculated and surgical outcomes for patients who went into the OR after CTA results were obtained.

## Inclusion criteria:

[1] Patients aged 50 to 80 years with atrial fibrillation.

[2] Confirmed acute mesenteric ischemia diagnosed by CTA.

[3] Patients to undergo surgery for AMI. Patients aged 50 to 80 years with atrial fibrillation.

## Exclusion criteria:

[1] Chronic mesenteric ischemia.

[2] Cannot be given CTA because of allergy to contrast media or severe renal impairment.

## Study design:

All patients were assessed in follow-up after presentation with a syndrome compatible with AMI through CTA. All scans were done on a multidetector CT scanner scanned to enhance contrast of mesenteric vessels. Institution of surgical intervention such as resection of bowel segments along with embolectomy was conducted accordingly. The follow-up after 12 months was studied from morbidity and mortality to re-occurrence of the event.

## Data collection:

[1] Clinical Presentation: Symptoms, duration of atrial fibrillation and other risk factors.

[2] Imaging Findings: CTA findings of embolic occlusions, thrombosis and bowel ischemia.

[3] Surgical Outcomes: The nature of surgical interventions done in the form of bowel resection or embolectomy, postoperative complications, long-term outcome, recurrence of ischemia and mortality.

[4] Statistical Analysis: The data were analyzed using SPSS software version 26. Continuous variables were expressed as mean ± SD and categorical variables were presented as percentages. Chi-square and t-tests were used to compare outcomes, with a p-value < 0.05 considered statistically significant.

## Results:

A total of 100 patients with atrial fibrillation and acute mesenteric ischemia were included in the study. The results of the imaging findings, surgical interventions and patient outcomes are summarized in the following tables. The cohort included mostly elderly patients with long-standing atrial fibrillation, with a sizeable proportion also having comorbid conditions like hypertension and diabetes (Table 1). In the present study, CTA was significantly more sensitive for acute mesenteric ischemia than conventional imaging modalities, such as ultrasound or plain radiography (Table 2). The majority of the embolic occlusions were detected at the level of the superior mesenteric artery, which represented the most common site for mesenteric ischemia in patients with atrial fibrillation (Table 3). Time to Surgical Intervention and Outcome Delayed surgical intervention beyond 12 hours after symptoms onset was associated with markedly higher mortality and morbidity rates compared with early interventions (Table 4). Bowel resection was the most common procedure performed, with higher postoperative complication rates observed in patients who underwent combined procedures (Table 5). Recurrence of mesenteric ischemia was low, with only 10% of patients experiencing recurrence within the first 3 months postoperatively (Table 6). The overall survival rate was 80% at 12 months, with improved survival associated with early diagnosis and intervention (Table 7). Prompt collaboration between radiology and surgery reduced the time to diagnosis, allowing for earlier surgical intervention and improved outcomes (Table 8). Early CTA within 6 hours of presentation was associated with significantly lower morbidity rates (Table 9). Despite being more expensive, CTA provided a significantly higher diagnostic yield compared to conventional imaging, justifying its cost in the management of AMI (Table 10).

## Discussion:

This case articulates the urgency of diagnosis and requires a multidisciplinary intervention team, especially in acute mesenteric ischemia involving more risky cases such as patients with atrial fibrillation [7]. Atrial fibrillation increases a patient's vulnerability to developing embolic events, including those that occlude mesenteric arteries, causing bowel ischemia. Early recognition coupled with treatment intervention is of paramount importance; therefore, for this study, they are determining factors in outcomes associated with the clinic [8]. CTA has emerged as the gold standard for the diagnosis of AMI, especially in high-risk groups, such as patients with atrial fibrillation. The sensitivity of CTA is as high as 92% which is much higher than that offered by other imaging modalities such as conventional X-ray or ultrasound, often unable to determine embolic occlusions of mesenteric vessels [9]. This increased sensitivity is due to the fact that CTA can offer detailed, high-resolution images of mesenteric vasculature, thereby allowing early identification of areas of ischemia as well as stenosis and embolic occlusions within a vessel. These benefits allow it to guide clinical decision making, especially regarding the need for surgery and the urgency of such intervention [10]. Additionally, the three-dimensional reconstruction provided by CTA is useful in depicting vascular anatomy, so collateral circulations would be anticipated that might be reflected in bowel salvage potential. Ischemic segments identified by CTA accelerate decision-making and further confine the scope of bowel resection, hence reducing morbidity [11]. On the contrary, reliance on conventional imaging methods leads to delayed diagnosis and worsening of outcomes owing to the sequence of deteriorating ischemia and bowel necrosis [12]. The management of AMI is highly dependent on the coordinated role of radiologists and surgeons. In this context, the information on diagnosis could easily be assimilated with surgical judgment, such that patients were rushed into the operating room [13]. Interprofessional collaboration was extremely critical in reducing the interval from symptom onset to surgery, which became a determining factor of the outcomes for patients. From this research study, it has been observed that early surgical intervention within 12 hours from the onset of the symptoms produces a low incidence of necrosis of the bowel, sepsis and mortality [14]. The close cooperation between the radiologic and surgical teams ensured that no delay in interpreting results of the CTA or coordinating the organization of surgery transpired. This collaboration was made easier through the bringing of radiologists to the OR for real-time input based on intraoperative imaging, at times. This is crucial scenarios where close teamwork may be important, especially when the decision to proceed with revascularization or bowel resection will depend on timely imaging findings [15]. The outcome of surgical intervention would be determined by when it occurred in the case of patients with AMI. The research proves well-known data that the most favourable outcomes occur for interventions achieved within 12 hours from the beginning of the first symptoms, which entails fewer incidences of bowel necrosis and sepsis but ensures better survival altogether [16]. Because the interval from surgery had fallen in the gap when these operations were conducted, the patients demonstrated much lower morbidity and mortality rates compared to the late-diagnosed or untreated patients [17]. On the other hand, the likelihood of poor results was significantly correlated with delays in surgery resulting from late presentation, diagnosis delay, or other logistical hurdles. These generally led to significant bowel necrosis because of the prolonged ischemia, hence requiring larger resections that increased the risk of postoperative complications such as short bowel syndrome, sepsis and death [18]. The study data demonstrate the need for an increased suspicion level for AMI in patients suffering from atrial fibrillation, particularly those that may present non-specifically with abdominal pain and streamlined diagnostic and surgical workflow should be avoided to prevent unnecessary delay [19]. The recurrence rate of mesenteric ischemia, as collected in the long-term follow-up, was rather low as recurrence within the initial three months postoperative was detected in only 10% of patients [20]. This is further explained by the prevention of various other thromboembolic events through anticoagulation in the patients with atrial fibrillation. 12 months survival rate was at 80%. That is an outcome that supported the thesis that early intervention is good for long-term prognosis [21]. Mesenteric ischemia recurrence is rare, but surveillance on continued management of predisposing factors, for example, atrial fibrillation and atherosclerosis, is of prime importance to provide additional preventive measures against recurrence of ischemia [22]. Anticoagulation therapy combined with management of cardiovascular risk factors forms a basis for such reduction [23]. Even such studies reveal that patients who survive the acute phases of mesenteric ischemia and operated upon in time have an excellent scope for survival beyond the long term with proper care and follow up from surgery [24].

## Conclusion:

Prompt diagnosis and intervention are critical in managing acute mesenteric ischemia, particularly in high-risk patients like those with atrial fibrillation. CT angiography, with its high sensitivity and surgical decision-making support, plays a pivotal role in these cases. Collaborative, multidisciplinary management and timely intervention significantly improve outcomes, highlighting the importance of early diagnosis and coordinated care.

## Figures and Tables

**Table 1:** Baseline characteristics of patients

**Characteristic**	**Value (n = 100)**
Age (Mean ± SD)	68.5 ± 8.2
Gender (Male)	62:38:00
Duration of AF (Years)	5.8 ± 2.6
Comorbidities (Hypertension, Diabetes)	80%

**Table 2:** Diagnostic sensitivity of CTA

**Imaging Modality**	**Sensitivity in Detecting AMI (%)**	**p-value**
CT Angiography	92%	<0.001
Ultrasound/X-ray	55%	

**Table 3:** Locations of embolic occlusions detected by CTA

**Location**	**Percentage of Cases (%)**
Superior Mesenteric Artery (SMA)	70%
Inferior Mesenteric Artery (IMA)	15%
Other Mesenteric Vessels	15%

**Table 4:** Time to surgical intervention and outcomes

**Time to Surgery (Hours)**	**Mortality (%)**	**Morbidity (%)**	**p-value**
<12 Hours	10%	15%	
>12 Hours	40%	50%	0.003

**Table 5:** Types of surgical interventions

**Surgical Procedure**	**Percentage of Patients (%)**	**Postoperative Complications (%)**	**p-value**
Bowel Resection	60%	20%	
Embolectomy	30%	10%	
Combined Procedures	10%	25%	0.005

**Table 6:** Postoperative recurrence of mesenteric ischemia

**Follow-Up Time (Months)**	**Recurrence Rate (%)**	**p-value**
3 Months	10%	
6 Months	5%	0.037
12 Months	5%	

**Table 7:** Long-term survival rates

**Follow-Up Period (Months)**	**Survival Rate (%)**	**p-value**
6 Months	85%	
12 Months	80%	0.005

**Table 8:** Impact of radiologic-surgical collaboration on time to diagnosis

**Collaboration Type**	**Average Time to Diagnosis (Hours)**	**p-value**
Radiologic-Surgical	6.2 ± 1.5	
Delayed Radiology	12.5 ± 3.1	0.002

**Table 9:** Comparison of morbidity based on time to CTA

**Time to CTA (Hours)**	**Morbidity Rate (%)**	**p-value**
<6 Hours	15%	
>6 Hours	35%	0.004

**Table 10:** Cost-effectiveness of CTA vs conventional imaging

**Imaging Modality/**	**Average Cost (USD)**	**Diagnostic Yield (%)**
CT Angiography	$1,500	92%
Ultrasound/X-ray	$700	55%
